# Cardiovascular Complications of COVID-19 Disease: A Narrative Review

**DOI:** 10.3390/diseases13080252

**Published:** 2025-08-08

**Authors:** Andrea Denegri, Valeria Dall’Ospedale, Marco Covani, Michal Pruc, Lukasz Szarpak, Giampaolo Niccoli

**Affiliations:** 1 Division of Cardiology, Parma University Hospital, 43126 Parma, Italy; 2 Division of Cardiology, University of Parma, Parma University Hospital, 43126 Parma, Italy; 3 Research Unit, Polish Society of Disaster Medicine, 05-806 Warsaw, Poland; 4 Henry JN Taub Department of Emergency Medicine, Baylor College of Medicine, Houston, TX 77030, USA; 5 Institute of Medical Science, Collegium Medicum, The John Paul II Catholic University of Lublin, 20-950 Lublin, Poland; 6 Department of Clinical Research and Development, LUXMED Group, 02-678 Warsaw, Poland

**Keywords:** COVID-19, cardiovascular complications, arrythmias, acute coronary syndrome, cardiac arrest, heart failure, Takotsubo cardiomyopathy, myopericarditis, right ventricular disfunction

## Abstract

Background: The coronavirus disease 2019 (COVID-19), caused by SARS-CoV-2, has had a profound impact on global health, extending beyond pulmonary complications. Cardiovascular involvement in COVID-19 is multifactorial and may be influenced by viral load, inflammatory response, and pre-existing comorbidities. Discussion: Acute complications include myocardial injury, arrhythmias, acute coronary syndromes (ACS), heart failure, Takotsubo cardiomyopathy, myopericarditis, and cardiac arrest. Notably, atrial fibrillation (AF) emerges as a frequent arrhythmic complication, particularly among critically ill patients, and is associated with increased mortality. COVID-19-patients with concomitant ACS present more severe clinical profiles and higher rates of thrombotic events, including stent thrombosis. Cardiac arrest predominantly presents with non-shockable rhythms and is associated with dismal outcomes. COVID-19 also exacerbates heart failure, both by aggravating existing cardiac dysfunction or by precipitating de novo heart failure. Takotsubo cardiomyopathy and myocarditis, although less frequent, have been reported and are often underdiagnosed due to subtle clinical presentations. Right ventricular dysfunction, linked to pulmonary involvement, has emerged as a key prognostic marker. Post-COVID-19 syndrome include persistent cardiac abnormalities such as reduced ventricular function and myocardial inflammation. Cardiac magnetic resonance imaging and strain echocardiography have proven useful in identifying subclinical cardiac involvement. Conclusions: Early recognition and monitoring of cardiovascular complications are crucial for improving outcomes in patients affected by COVID-19. This review summarizes current evidence regarding cardiovascular manifestations associated with COVID-19.

## 1. Introduction

Severe acute respiratory syndrome coronavirus 2 (SARS-CoV 2), or coronavirus disease 2019 (COVID-19), virus pandemic has drastically changed the health service and has paralyzed medical services [1]. Recent growing evidence suggests that COVID-19 can disrupt several systems and results in a variety of clinical symptoms. The mechanisms behind how COVID-19 affects the cardiovascular system are currently being discovered. The size of the viral inoculum, the intensity of the host immune response, and the existence of underlying comorbidities may all influence the cardiovascular involvement in COVID-19, and frequently, these patients present newly discovered or worsening cardiovascular disease, hypertension, arrhythmia, myocardial infarction, heart failure, and sudden cardiac arrest [1,2,3,4,5,6]. Post-COVID-19 syndrome is a public health concern that has emerged as a result of the COVID-19 pandemic, and has long-lasting impacts on those who have already contracted COVID-19. The Royal College of General Practitioners (RCGP) has thus divided COVID-19 infection into three timeframe points: signs and symptoms that last for up to four weeks are referred to as acute COVID-19, those that last for four weeks to twelve weeks are referred to as ongoing symptomatic COVID-19, and those that last for more than twelve weeks are referred to as post-COVID-19 syndrome. Since it affects COVID-19 survivors at all illness severity levels, post-COVID-19 syndrome is a poorly known condition. The condition is mostly characterized by post-discharge tiredness and dyspnea [7,8]. The need to determine the persistent symptoms and clinical consequences has been driven by the limited understanding of its clinical course. The aim of this review was to explore the most common cardiovascular (CV) complications of COVID-19.

## 2. Cardiovascular Manifestations of COVID-19

**Figure 1:**
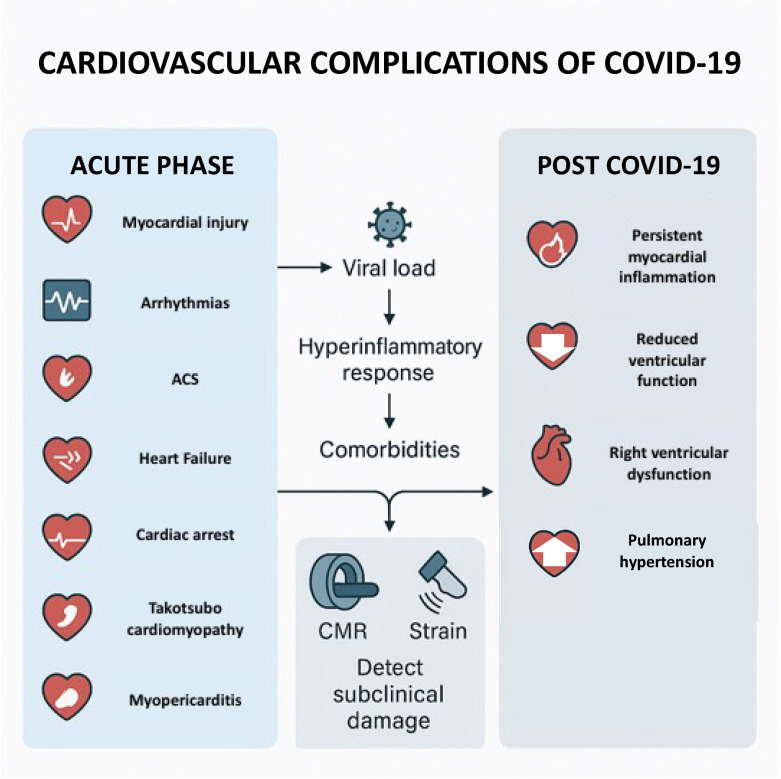
Illustrative figure of acute and long-term COVID-19 manifestations.

SARS-CoV-2, the virus responsible for COVID-19, is now recognized not only as a respiratory pathogen, but also as a significant threat to cardiovascular health. Beyond the acute phase of infection, a substantial proportion of patients experience a range of cardiovascular complications, contributing to both short-term morbidity and long-term sequelae. The mechanisms underlying these complications are multifactorial, involving direct viral myocardial injury, systemic inflammation, endothelial dysfunction, hypercoagulability, and heightened metabolic stress on the cardiovascular system. Common acute cardiovascular manifestations include myocarditis, pericarditis, arrhythmias (such as atrial fibrillation and ventricular tachycardias), acute coronary syndromes (due to plaque instability or thrombotic events), and exacerbation of pre-existing cardiovascular conditions like heart failure and hypertension (Figure 1). The diagnosis and risk stratification of cardiovascular involvement in COVID-19 rely on a combination of clinical assessment, laboratory biomarkers, and imaging modalities [9].

Electrocardiography (ECG) is a readily available and fundamental tool for detecting acute myocardial injury, ischemia, new-onset arrhythmias, conduction abnormalities, ST-T segment deviations, or QT prolongation that can provide crucial early indicators of cardiac distress [10].

Cardiac biomarkers, particularly high-sensitivity troponin and N-terminal pro-B-type natriuretic peptide (NT-proBNP), are indispensable for identifying myocardial injury and ventricular dysfunction, respectively. High troponin levels correlate with an increased risk of adverse outcomes, including mortality, while elevated NT-proBNP suggests cardiac strain and potential heart failure development [11].

Imaging techniques play a pivotal role in characterizing the extent and nature of cardiovascular damage. Echocardiography assesses ventricular function, valvular abnormalities, pericardial effusions, and pulmonary hypertension, identifying subtle signs of myocardial dysfunction, even in patients with preserved ejection fraction, and can guide management decisions [12,13].

Cardiac computed tomography (CT) can be utilized to evaluate coronary arteries, assess for pulmonary embolism, and detect pericardial effusions. COVID-19 patients are at increased risk for future CV events and in this setting coronary CT has demonstrated a more rapid progression of coronary atherosclerosis with increased risk of target lesion failure [14].

On the other hand, CMR can precisely delineate areas of myocarditis, identify myocardial scarring, and provide insights into diffuse myocardial injury that might be missed by other modalities. The multi-parametric capabilities of CMR make it particularly useful both in the acute and post-acute phase, although the COVID-19 pandemic has determined a disproportion between the number of CMR requested and positive results [15].

The cardiovascular implications for outcomes in COVID-19 patients are severe. Acute cardiac injury and the development of major adverse cardiovascular events (MACE) during hospitalization significantly increase the risk of in-hospital mortality [16]. Beyond the acute phase, a considerable number of individuals, including those with mild initial infections, experience “Long COVID” or Post-Acute Sequelae of SARS-CoV-2 infection (PASC), with persistent cardiovascular symptoms such as palpitations, chest pain, and exercise intolerance [17]. This suggests a long-term burden on cardiovascular health, necessitating ongoing monitoring and potential intervention. The cumulative impact of direct viral effects, systemic inflammation, and prolonged recovery can lead to a significant increase in the prevalence of cardiovascular morbidity and mortality in the years following the pandemic. Therefore, a multidisciplinary approach encompassing early diagnosis, risk stratification, and long-term cardiovascular follow up is crucial to mitigate the lasting impact of COVID-19 on global cardiovascular health.

### 2.1. Arrhythmias

Cardiac arrhythmias represent one of the most thoroughly investigated cardiovascular complications in patients with COVID-19. Among these, atrial fibrillation (AF) is the most frequently reported arrhythmia, and is independently associated with increased morbidity and mortality across various clinical settings [18].

Initial evidence during the early stages of the pandemic highlighted an elevated incidence of AF in COVID-19 patients, particularly among those with severe disease. In one early observational study conducted in Wuhan, China, AF was observed more frequently in hospitalized COVID-19 patients compared to those with bacterial pneumonia [19]. Conversely, a case–control analysis revealed a paradoxically lower incidence of new-onset AF in critically ill COVID-19 patients undergoing mechanical ventilation compared to those with non-COVID-19 pneumonia (10% vs. 30%) [20]. No significant differences were found in the incidence of ventricular tachycardia (VT) or bradyarrhythmia between the two groups. A large-scale analysis from the Southeastern Michigan COVID-19 Consortium Registry Database (SMCRD registry), involving 6927 COVID-19 patients and 14,174 influenza patients, reported a 9% incidence of new-onset atrial arrhythmias (AAs) in the COVID-19 cohort, associated with significantly worse clinical outcomes, including increased requirements for mechanical ventilation, vasopressor support, and an all-cause in-hospital mortality of 39.6%, compared to 6.3% in the influenza cohort [21]. Similarly, an observational study involving 3970 COVID-19 patients and 1420 influenza patients showed that AF/AFL was more common in COVID-19 (13%), though new-onset events were relatively infrequent (3.7%). In both groups, new-onset arrhythmias were associated with elevated levels of inflammatory markers (e.g., IL-6, CRP), myocardial injury (elevated troponin), and prothrombotic biomarkers (D-dimer), suggesting an inflammation-mediated mechanism [22].

Inflammation has emerged as a central contributor to the pathophysiology of arrhythmogenesis in COVID-19. Elevated levels of pro-inflammatory cytokines, such as IL-6 and IL-10, have been significantly associated with the development of both atrial and ventricular arrhythmias [23,24]. These findings are corroborated by recent studies that further link systemic inflammatory burden with electrical instability and atrial remodeling in SARS-CoV-2 infection [25]. Beyond intrinsic inflammatory mechanisms, certain pharmacologic agents used during the early pandemic phase—most notably hydroxychloroquine and azithromycin—were hypothesized to increase arrhythmic risk. However, retrospective analyses failed to demonstrate a significant rise in arrhythmia incidence with these agents that should also be reconsidered in COVID-19 management protocols [26,27].

Epidemiologic studies consistently identify advanced age, hypertension, and pre-existing cardiovascular disease as independent predictors of AF occurrence in hospitalized COVID-19 patients [9,28]. New-onset AF has also been linked to prolonged hospital stays and more severe disease courses and has been associated with worse clinical outcomes. A multicenter analysis showed a threefold increase in mortality risk among patients with arrhythmias compared to those without (25.9% vs. 0.1%, *p* < 0.001) [29]. These patients also exhibited higher rates of ICU admission and mechanical ventilation. Notably, atrial fibrillation was independently associated with both increased mortality and the risk of life-threatening ventricular arrhythmias [30]. Evidence on bradyarrhythmias, atrioventricular blocks, and non-sustained ventricular tachycardia is more fragmentary and comes from small studies [31,32,33,34]. These observations are summarized in Table 1.

### 2.2. Acute Coronary Syndrome

Patients presenting with acute coronary syndrome (ACS) and concurrent SARS-CoV-2 infection exhibit a more complex clinical phenotype, characterized by a higher prevalence of CV risk factors and a significantly increased incidence of adverse in-hospital outcomes. ST-segment elevation myocardial infarction (STEMI) is the most commonly observed ACS subtype in this population, reflecting a more severe thrombotic and inflammatory burden [35,36,37]. Patients with COVID-19-related ACS have a more complex history of CV comorbidities more frequently, including hypertension, diabetes mellitus, chronic kidney disease, and established ischemic heart disease, when compared with non-COVID ACS cohorts. Clinically, they present with more severe manifestations at admission, with a notably higher incidence of acute heart failure and cardiogenic shock [38]. These findings suggest a synergistic interaction between systemic inflammation and myocardial stress in SARS-CoV-2 infection, which may exacerbate myocardial oxygen demand–supply mismatch and contribute to hemodynamic instability.

Coronary angiographic data showed a higher prevalence of multivessel disease and predominant involvement of the left anterior descending artery in patients with COVID-19 and ACS. Notably, rates of stent thrombosis in this cohort have been reported around 20%, substantially exceeding historical rates in non-COVID populations [39]. This phenomenon may be attributable to the prothrombotic milieu induced by SARS-CoV-2, characterized by endothelial dysfunction, platelet activation, and hypercoagulability.

Longitudinal outcome data reveal that subjects with concomitant COVID-19 and ACS face a disproportionately high risk of adverse events beyond the acute phase. Specifically, these patients demonstrate elevated 90-day post-discharge mortality (odds ratio 2.09) and significantly increased one-year mortality rates (21.1% vs. 6.5%) compared to ACS patients without SARS-CoV-2 infection [40]. Nevertheless, acute myocardial infarction during active SARS-CoV-2 infection remains associated with increased rates of major complications, including vasopressor requirement, mechanical ventilation, and intensive care unit admission [41,42]. These findings emphasize the necessity of post-discharge surveillance and risk modification strategies in this high-risk population.

### 2.3. Heart Failure

**Figure 2:**
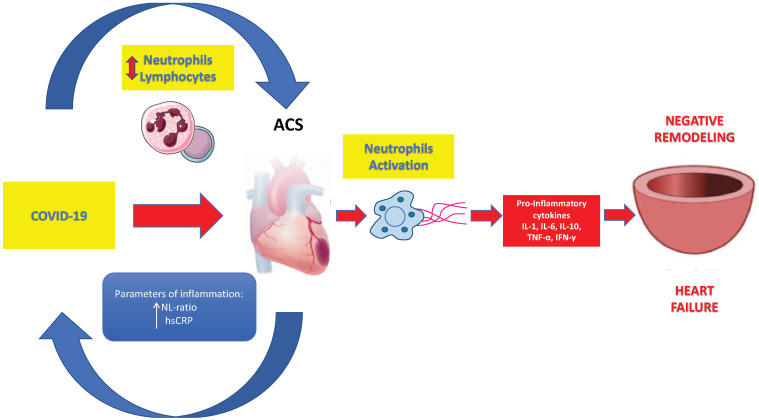
COVID-19 and HF: the multifactorial interplay, involving direct myocardial injury, systemic inflammation, endothelial dysfunction, and alterations in hemodynamic load.

Heart failure (HF) has emerged as a critical comorbidity that significantly influences clinical outcomes in patients with SARS-CoV-2 infection. The pathophysiological interplay between COVID-19 and HF is multifactorial, involving direct myocardial injury, systemic inflammation, endothelial dysfunction, and alterations in hemodynamic load (Figure 2).

These mechanisms can exacerbate pre-existing cardiac dysfunction or precipitate acute heart failure (AHF) in previously stable or undiagnosed individuals. In a large observational study involving 3080 patients with confirmed COVID-19, 4.9% had a history of chronic HF [43]. Among them, the incidence of AHF during hospitalization was substantially higher than in patients without a prior HF diagnosis (11.2% vs. 2.1%; *p* < 0.001), and this group exhibited markedly elevated in-hospital mortality (48.7% vs. 19.0%; *p* < 0.001). Notably, 2.5% of patients without prior HF developed de novo AHF, suggesting a potential direct role of SARS-CoV-2 infection in unmasking or inducing acute cardiac decompensation.

Further evidence reinforces the association between HF and poor outcomes in COVID-19. A comparative analysis reported significantly greater in-hospital mortality in COVID-19 patients with a history of HF (36% vs. 23%; *p* < 0.001), with an odds ratio of 1.93 for mortality [44]. These patients also demonstrated a higher likelihood of requiring intensive care unit admission (33% vs. 21%) and non-invasive ventilatory support (48% vs. 30%). Coexisting conditions such as atrial fibrillation (AF) and chronic kidney disease (CKD) were independently associated with increased risk for AHF events.

In critically ill COVID-19 patients, the burden of AHF remains significant. One multicenter cohort study reported AHF incidence of 8.9% among hospitalized patients with severe COVID-19, most of whom had underlying HF, AF, or CKD [45]. In-hospital mortality in those who developed AHF was notably higher (43.8% vs. 32.4%; *p* = 0.040), suggesting a synergistic role of pre-existing cardiovascular comorbidities in driving adverse outcomes.

COVID-19-infected individuals with HF are at heightened risk of developing severe complications, including acute respiratory distress syndrome (ARDS), multiorgan failure, and the need for mechanical ventilation and vasopressor support [46]. These findings support the need for proactive management and resource planning for this vulnerable group [47]. Moreover, evidence from recent cohorts suggests that patients with HF are not only more susceptible to early mortality but may also face increased risks in post-acute phases of COVID-19, particularly those with reduced ejection fraction [48].

Taken together, these data highlight HF as a major determinant of disease severity in COVID-19. Early identification and personalized management strategies are critical to improving outcomes in this high-risk population.

### 2.4. Right Ventricular Dysfunction and Pulmonary Hypertension

Right ventricular (RV) dysfunction has emerged as a significant cardiovascular complication in patients affected by COVID-19. RV impairment is strongly associated with adverse clinical outcomes, including elevated morbidity and mortality, particularly in patients with severe respiratory involvement and those requiring intensive care [49].

Comparative echocardiographic studies have demonstrated that RV dysfunction may be more prevalent than left ventricular (LV) impairment in the post-acute phase of COVID-19. In a cohort of recovered patients, RV dysfunction was reported in over one-third of cases, compared to less than one-fifth exhibiting LV dysfunction [50].

Standard echocardiographic indices may underestimate RV dysfunction in severe COVID-19, especially in patients requiring extracorporeal membrane oxygenation (ECMO). More advanced parameters—such as RV fractional area change (FAC) and velocity-time integral (VTI)—have shown superior sensitivity, identifying RV dysfunction in 72–86% of patients versus 23% using TAPSE alone [51]. These findings highlight the necessity of multimodal imaging strategies to accurately characterize RV performance.

Longitudinal data further reinforce the prognostic importance of RV involvement. RV dysfunction has been independently linked to increased one-year mortality following COVID-19 hospitalization, particularly among older adults and those with underlying cardiovascular disease or reduced LV ejection fraction [52]. Conversely, recovery of RV function correlates with improved respiratory outcomes and overall survival, suggesting potential reversibility with effective clinical management [53].

Persistent RV dysfunction and pulmonary hypertension have also been documented as long-term sequelae of COVID-19, even in individuals without preexisting cardiopulmonary disease. While these abnormalities tend to regress during convalescence, they may persist in a subset of patients, warranting long-term surveillance and cardiovascular follow up [54].

### 2.5. Takotsubo Cardiomyopathy

Takotsubo cardiomyopathy (TTC), or stress-induced cardiomyopathy, has been increasingly reported in association with SARS-CoV-2 infection. Although its precise incidence remains difficult to ascertain, echocardiographic studies indicate a low but notable prevalence among hospitalized patients. In a large observational study, apical ballooning—characteristic of TTC—was observed in approximately 0.7% of individuals with confirmed COVID-19 [55].

The psychosocial and physiological stress induced by the pandemic, compounded by direct viral and inflammatory myocardial insults, are hypothesized to contribute to the pathogenesis of TTC in this setting. Despite reports of increased stress burden during the pandemic, the overall incidence of TTC does not appear to have increased significantly [56]. Nevertheless, COVID-19-associated TTC has been linked to heightened risk of complications, including cardiogenic shock, acute kidney injury, and in-hospital mortality [57].

Current evidence underscores the need for more granular, prospective data to elucidate the prevalence, clinical characteristics, and long-term implications of TTC triggered by COVID-19 [58]. The heterogeneity of clinical presentations and overlap with other forms of acute cardiac injury complicate diagnosis, and highlight the necessity of imaging confirmation and biomarker correlation.

### 2.6. Myopericarditis

Myocarditis and pericarditis are increasingly recognized as inflammatory cardiac complications of SARS-CoV-2 infection. Myocardial inflammation may be subclinical or overt, presenting with chest pain, dyspnea, or hemodynamic instability. In a study of patients with biopsy- or cardiac MRI-confirmed COVID-19 myocarditis, the median patient age ranged from 38 to 47 years, with a mild male predominance [59]. Common presenting symptoms included chest pain (55%) and dyspnea (54%), though nearly 60% of cases lacked radiographic evidence of pneumonia.

COVID-19 myocarditis’s prevalence varies widely. Autopsy studies suggest a rate of 7.5%, while cardiac MRI investigations in athletic populations have reported myocarditis in 2.3% of infected individuals, with most cases being asymptomatic [60]. Subclinical myocardial inflammation, therefore, may be more common than previously assumed, potentially contributing to post-acute sequelae or cardiac dysfunction [61].

Patients with COVID-19 myocarditis are more likely to have pre-existing cardiovascular risk factors and experience more severe disease. Hemodynamic instability is frequent, with nearly 40% of hospitalized cases requiring vasopressor support or mechanical circulatory assistance [62]. Mortality among these patients can reach 16%, and when compared to non-COVID-19 myocarditis, SARS-CoV-2-related myocarditis confers a significantly higher risk of adverse outcomes (OR = 7.75; 95% CI = 2.77–21.7) [63]. Notably, absence of pneumonia has been associated with more favorable outcomes, suggesting that pulmonary co-involvement may exacerbate myocardial injury.

Pericarditis has similarly been documented in a large COVID-19 cohort, with an incidence of approximately 1.5%. It is associated with an increased risk of cardiac arrest, heart failure, atrial fibrillation, and myocardial infarction, with a six-month mortality exceeding that of non-pericarditis patients [64].

Finally, myopericarditis has been reported as a rare complication of mRNA-based COVID-19 vaccination, particularly among adolescent males following the second dose. A meta-analysis has shown no significant difference in risk between pediatric and adult populations, although adolescents and males constitute the highest-risk subgroups [65].

### 2.7. Cardiac Arrest

In-hospital cardiac arrest (IHCA) has emerged as a serious complication in patients hospitalized with COVID-19, particularly during the early phases of the pandemic. Initial observational studies from Wuhan revealed that the majority of COVID-19-related IHCA events stemmed from respiratory causes (87.5%), with asystole as the predominant rhythm (up to 90%) and a low incidence of shockable rhythms (~5.9%) [66]. Return of spontaneous circulation (ROSC) was achieved in only 13.2% of patients undergoing cardiopulmonary resuscitation (CPR), with a 30-day survival rate of merely 2.9%, highlighting the extremely poor prognosis in this population.

IHCA occurred in approximately 7.5% of hospitalized COVID-19 patients, with a predominance of asystole (69.2%) and an alarming in-hospital mortality rate approaching 97% [67]. A large-scale analysis from the American Heart Association COVID-19 Cardiovascular Disease Registry (n = 8518) identified IHCA in 5.9% of patients. The risk was disproportionately higher in ICU patients and those of older age, Hispanic or Black race, and individuals with pre-admission oxygen dependence [68].

Among critically ill COVID-19 patients, IHCA incidence has ranged from 14% to 18%, with non-shockable rhythms (primarily asystole and pulseless electrical activity) observed in the majority [69,70]. These rhythms correlate with significantly lower survival and support the hypothesis that COVID-related IHCA often arises from severe respiratory or metabolic derangements rather than primary cardiac events. Comparative analyses have shown that while COVID-19 patients require higher levels of advanced support—such as vasoactive agents, mechanical ventilation (76.4% vs. 23.6%; *p* < 0.001), and renal replacement therapy (18.2% vs. 3.6%; *p* = 0.029)—their in-hospital cardiovascular outcomes, when adjusted, are not significantly worse than non-COVID IHCA cases [71]. Nevertheless, COVID-19 patients tend to be younger and more frequently present with non-shockable rhythms (90.5% vs. 84.0%; *p* < 0.001), increased use of inotropes (39.2% vs. 29.6%; *p* < 0.001), and lower survival to discharge (11.9% vs. 23.5%; *p* > 0.001) [72,73].

A consistent theme across studies is the markedly reduced likelihood of ROSC and 30-day survival in COVID-19-associated IHCA, suggesting a more severe systemic illness with complex multi-organ dysfunction as the predominant mechanism [74]. This pattern supports the conclusion that IHCA in COVID-19 is often the culmination of multi-factorial pathophysiological deterioration rather than an isolated cardiac event.

## 3. Post-COVID-19 Cardiovascular Complications

The persistence of cardiovascular complications following recovery from SARS-CoV-2 infection has become a major concern. Dyspnea, fatigue, chest discomfort, and reduced exercise capacity are the main persistently symptoms after the recovery from the acute phase of COVID-19, and these may be related to RVD or PH, that are probably underestimated in post-COVID-19 patients [54].

Increasing evidence from echocardiography [50], cardiac magnetic resonance (CMR) [75], and biomarker studies [76] points to subclinical myocardial injury in both hospitalized and non-hospitalized individuals. However, the clinical relevance of these findings remains a subject of ongoing investigation.

Echocardiographic data indicate that up to 36.8% of post-COVID patients exhibit right ventricular dysfunction, with 17.9% showing impaired global left ventricular function and 25.5% developing pericardial effusions [50]. These abnormalities are frequently subclinical and may not manifest with overt symptoms. Strain imaging studies have shown that global longitudinal strain (GLS) may remain preserved, but segmental strain abnormalities—particularly in basal segments—can persist and reflect localized injury [77]. Similarly, global circumferential strain (GCS) abnormalities in both ventricles have been linked to prior inflammation and predict adverse remodeling, even in the absence of symptomatic heart failure.

Advanced myocardial tissue characterization via CMR has demonstrated the presence of late gadolinium enhancement (LGE) in 30–54% of patients several weeks to months after acute illness, particularly in those with elevated cardiac biomarkers such as high-sensitivity troponin I (hs-TnI) [78].

Among patients with non-severe disease, clinical assessments within 2–3 weeks post-discharge revealed new electrocardiographic changes (e.g., T-wave inversions), sinus bradycardia, and biochemical evidence of myocardial injury in 42.3% of cases [79,80]. One prospective analysis found no significant differences in symptoms, ECG, or health-related quality of life between patients with and without CMR evidence of injury, despite marked differences in LGE prevalence and troponin levels [76]. This raises important questions about the clinical consequences of subclinical myocardial involvement and whether these findings portend future cardiovascular events.

While evidence clearly indicates the presence of myocardial involvement after SARS-CoV-2 infection, the long-term consequences of LGE, strain abnormalities, and elevated biomarkers are still unclear, particularly in asymptomatic individuals. Moreover the role of persistent inflammation, endothelial injury, or autoimmune phenomena potentially responsible of chronic myocardial changes is still under investigation. Finally, from a practical point of view, there is no established protocol to stratify patients at risk for post-COVID cardiac complications, especially those who were not hospitalized. In this setting, cardiac MRI and strain imaging offer invaluable diagnostic insight, but their application must be balanced against clinical utility and cost-effectiveness.

## 4. Conclusions

Patients with pre-existing cardiovascular disease exhibit heightened vulnerability to adverse outcomes both during acute and chronic SARS-CoV-2 infection. The timely recognition of cardiovascular involvement through comprehensive clinical evaluation, biomarker profiling, and appropriate imaging is critical to mitigate disease progression and optimize therapeutic strategies. The identification of prognostic indicators and high-risk subgroups will be essential for guiding targeted monitoring, preventive interventions, and personalized therapeutic approaches.

## Figures and Tables

**Table 1:** Atrial fibrillation in COVID-19 disease.

	Patients	Type	AF	VF	Mortality
Han K.Y. et al.	84	Hospitalized	4 (4.8%)	3 (3.6%)	nd
Rav-Acha M. et al.	390	Hospitalized	20 (5.1%)	2 (0.51%)	nd
Denegri A. et al.	637	Hospitalized	49 (7.7%)	nd	Increased
Bertini M. et al.	432	Critical	95 (22%)	nd	nd
Merino JL et al.	3416	Hospitalized	48 (3.2%)	9 (0.6%)	Increased
Peltzer B. et al.	1053	Hospitalized	166 (15.8%)	27 (2.6%)	Increased
Jirak P. et al.	60	Critical	6 (10%)	18 (30%)	Increased
Jehangir Q. et al.	6927	Hospitalized	626 (9%)	nd	Increased
Garciía-Granja P.E. et al.	517	Hospitalized	54 (10.4%)	nd	Increased
Musikantow D. R. et al.	3970	Hospitalized	146 * (3.7%)	nd	Increased
Guan H. et al.	463	Hospitalized	17 ** (3.7%)	1 (0.2%)	nd
Reynbakh O. et al.	59	Hospitalized	9 (15%)	15 (25%)	nd
Yarmohammadi H. et al.	1029	Hospitalized	46 * (4.5%)	nd	Increased
Parwani A. S. et al.	113	Critical	40 ** (35.4%)	35 (31%)	nd
Turagam M. K. El al.	140	Hospitalized	nd	7 (5%)	nd
Vijayabharathy K. et al.	109	Critical	16 (14.6%)	nd	Increased
Dagher L. et al.	310	Hospitalized	23 * (7.4%)	nd	Increased
Erguün B. et al.	248	Critical	37 (14.9%)	nd	Increased
Sano T. et al.	673	Hospitalized	28 (4.2%)	nd	Increased

* AF + AFL. ** Non-new-onset AF.
